# Characterization of the Symbiotic Nitrogen-Fixing Common Bean L*ow Phytic Acid* (*lpa1)* Mutant Response to Water Stress

**DOI:** 10.3390/genes9020099

**Published:** 2018-02-15

**Authors:** Remo Chiozzotto, Mario Ramírez, Chouhra Talbi, Eleonora Cominelli, Lourdes Girard, Francesca Sparvoli, Georgina Hernández

**Affiliations:** 1 Center for Genomic Sciences, National Autonomous University of Mexico, Av, Universidad 1001, Cuernavaca 62210, Mor., Mexico; remo.chiozzotto@gmail.com (R.C.); mario@ccg.unam.mx (M.R.); chouhra29@hotmail.com (C.T.); girard@ccg.unam.mx (L.G.); 2 Institute of Agricultural Biology and Biotechnology, National Research Council, IBBA-CNR, Via Edoardo Bassini 15, 20133 Milano, Italy; cominelli@ibba.cnr.it (E.C.); sparvoli@ibba.cnr.it (F.S.)

**Keywords:** common bean, legume-rhizobia interaction, *low phytic acid* mutant, response to water stress, symbiotic nitrogen fixation

## Abstract

The common bean (*Phaseolus vulgaris* L.) *low phytic acid* (*lpa1*) biofortified genotype produces seeds with improved nutritional characteristics and does not display negative pleiotropic effects. Here we demonstrated that *lpa1* plants establish an efficient nitrogen-fixing symbiosis with *Rhizobium etli* CE3. The *lpa1* nodules showed a higher expression of nodule-function related genes than the nodules of the parental wild type genotype (BAT 93). We analyzed the response to water stress of *lpa1* vs. BAT 93 plants grown under fertilized or under symbiotic N_2_-fixation conditions. Water stress was induced by water withholding (up to 14% soil moisture) to fertilized or *R. etli* nodulated plants previously grown with normal irrigation. The fertilized *lpa1* plants showed milder water stress symptoms during the water deployment period and after the rehydration recovery period when *lpa1* plants showed less biomass reduction. The symbiotic water-stressed *lpa1* plants showed decreased nitrogenase activity that coincides with decreased sucrose synthase gene expression in nodules; lower turgor weight to dry weight (DW) ratio, which has been associated with higher drought resistance index; downregulation of carbon/nitrogen (C/N)-related and upregulation of stress-related genes. Higher expression of stress-related genes was also observed in bacteroids of stressed *lpa1* plants that also displayed very high expression of the symbiotic *cbb_3_* oxidase (*fixN*d).

## 1. Introduction

Common bean (*Phaseolus vulgaris* L.) is the world’s most important food legume; it provides up to 15% of total daily calories and 36% of total daily proteins in different regions of Africa and Americas. As a legume, common bean can establish symbiosis with soil bacteria known as rhizobia to capture atmospheric N_2_ and thereby to support plant growth. The symbiotic nitrogen fixation (SNF) occurring in the legume-rhizobia symbiosis is the main source of N_2_ in agro-ecosystems. The common bean crop is grown by smallholder farmers in Latin America and East Africa, where it is often exposed to unfavorable conditions and minimum use of inputs [1,2,3].

About 60% of common beans produced worldwide are grown in regions subjected to water stress, thus, after diseases, drought is the second most important factor that contributes to yield reduction. Because of this, the development of common bean varieties with improved water stress tolerance has been a long-time major objective for breeding programs in different regions [4,5]. Drought tolerance in legumes, such as common bean or soybean, differs depending on the use of alternative N_2_ sources: nitrate in fertilized plants vs. fixed-N_2_ in SNF plants [6,7]. However, common bean breeding for drought-tolerance selection is performed mainly in nitrate-fertilized plants, thus neglecting possible tolerance increase in SNF plants [7]. Usually breeding programs for improving Mesoamerican and Andean common bean cultivars drought tolerance have based solely on grain yield and their contribution to crop improvement has been rather considered poor.

The SNF process is highly sensitive to adverse environmental conditions, including water deficit or drought, being N_2_-fixation rate one of the first physiological processes affected. Among other environmental disturbances, the impairment of SNF in response to drought is the sum of rhizobia and plant effects. Drought has a drastic negative effect on rhizobial colonization/infection, often related to a decrease in population levels of bacteria in the rhizosphere. This would result in partial or total inhibition of nodule development and function [8,9]. Several studies have been carried out to explain the decrease in N_2_-fixation rates under drought conditions. A direct effect on nodule oxygen permeability, which provokes O_2_ limitation to the bacteroid, and accumulation of N-compounds—either in the shoots or in the nodules and a nodular carbon flux shortage—have been related to the N_2_-fixation sensitivity under drought [8,10,11]. The importance of carbohydrate metabolism in water stressed nodules has been demonstrated; the nodule sucrose synthase activity decreases with a consequent decrease of the malate content, that is the main carbon source for bacteroid respiration, thus inducing functional disability of the bacteroid [12,13]. Reactive oxygen species (ROS) production in the nodule is an additional mechanism for the control of N_2_-fixation under drought, resulting in an imbalanced redox state of the nodule coupled to overexpression of catalase and isocitrate dehydrogenase [13].

Common bean seeds have a high content of essential minerals such as iron, zinc and calcium that are important in the diet, however these minerals are scarcely bioavailable. For this reason, this specie was chosen by the HarvestPlus program, an initiative of the Consultative Group for International Agricultural Research (CGIAR), as one of the target species to be iron biofortified [14]. Poor mineral bioavailability is caused mainly by the presence of high content of phytic acid (*myo*-inositol-1,2,3,4,5,6-hexa*kis*phosphate; InsP_6_) [15]. This compound is a strong cation chelator, stored as the main source of phosphorous (P) that becomes available in the developing seedlings due to the activity of phytases, enzymes that hydrolyze phytate into inorganic phosphate and lower *myo*-inositol phosphates (InsPs). These enzymes are not present in monogastric animals, including humans. One of the strategies proposed to increase the bioavailability of minerals is the development of *low phytic acid* (*lpa*) mutants [16], which have been obtained for several plant species including the common bean *lpa1* mutant [17,18]. The *lpa1* mutation affects the *PvMRP1* (*P. vulgaris* multidrug resistance-associated Protein 1) gene, that, together with its paralog *PvMRP2,* encode putative vacuolar phytic acid transporter, belonging to the plant ATP-binding cassette (ABC) transporters cluster C (ABCC) [19]. In seeds *PvMRP1* is highly expressed, while *PvMRP2* expression is hardly detectable. Conversely, both of them are expressed at similar levels in different plant organs, including in nodules [20]. The *lpa1* seeds have several nutritionally important characteristics, such as a 90% reduction in phytic acid content, a 25% reduction of raffinosaccharides content, a 30% reduction of *myo*-inositol and a 7-fold increase of free iron cations that resulted to be more bioavailable [15,17,18]. Furthermore, a negative feedback on the expression of the *myo*-inositol phosphate synthase gene (*MIPS*) and other key genes of the phytic acid biosynthetic pathway was observed [18]. Besides its role as a P storage compound, phytic acid plays important roles in the regulation of different cell processes together with lower and higher InsPs [21], thus *lpa* mutants are often associated with negative pleiotropic effects on seed and plant performance [22,23,24]. Exceptions to these are the *lpa* mutant from barley (*lpa1-1)* that showed a good agronomic performance in both irrigated and non-irrigated environments [22], the *Arabidopsis* mutant *atmrp5* which is drought tolerant [25] and the common bean *lpa1* mutant that does not show negative pleiotropic effects on traits of agronomic relevance, such as seedling emergence, dry seed yield, seed weight and plant growth duration [16,17].

Projects aimed at obtaining *lpa* crops should take into account aspects regarding their agronomic potential. The ability of legume crops to develop symbiotic relationships with rhizobia is an advantage to perform in low input conditions. Although *lpa* mutants have been described also in other legumes, including soybean [26,27,28] and pea [29], to our knowledge no characterization of the performance of these mutants to SNF has been reported.

In this work, we analyzed the SNF performance of the common bean *lpa1* mutant plants, showing that it is capable of establishing functional rhizobial symbiosis similarly as its parental genotype BAT 93. In addition, we assessed the response to water stress of *lpa1* plants both in SNF and fertilized conditions.

## 2. Materials and Methods

### 2.1. Plant Material and Growth Conditions

The common bean (*P. vulgaris* L.) genotypes used in this study were the Mesoamerican BAT 93 genotype and its derived mutant *lpa1* and the original *lpa280-10* and its 905 sister line [17,30]. The original *lpa280-10* mutation, isolated through mutagenesis of the 905 genotype [18], was introgressed with three backcross cycles into the BAT 93 genotype [20]. Phenotypic analysis of the *lpa* trait was performed with the high inorganic phosphorous (HIP) assay that allows a vital screening of dry seeds [17], while a cleaved amplified polymorphic sequence (CAPS) molecular marker was developed for plantlet genotyping [20].

At the Center for Genomic Sciences, UNAM (CCG-UNAM, Cuernavaca, México), plants (wild type (wt) BAT 93 and *lpa1* BAT 93) were grown in growth chambers with a temperature/relative humidity of 27 ± 1 °C/50 ± 10% during a normal watering regime and 27 ± 1 °C/35 ± 5% during water withholding. Light was provided with an average intensity of 120 ± 20 µmol m^−2^ s^−1^ and a photoperiod of 16/8 h. For symbiotic conditions, surface sterilized seeds were germinated in sterile conditions for 2 days and then seedlings were transferred to pots (5 seedlings per pot) containing 3 kg of wet sterilized vermiculite. Plants were inoculated with 1 ml of a saturated culture (10^8^ cells mL^−1^) of the *Rhizobium etli* CE3 wt strain and were watered every two days with 250 mL (placed in a plate below each pot) of N_2_-free nutrient solution [31] and alternatively of deionized water. After three weeks under normal water regime, when inoculated plants showed active nodules, a set of pots were subjected to irrigation withholding up to 15 days to induce water stress. For fertilized (non-symbiotic) condition, plants were grown in sealed sterile pots (cardboard lid and sterile cotton around the stem) and watered similarly but using alternatively full-nutrient solution [31] or deionized water. Treatment for water stress was similar for fertilized plants. Soil water content was monitored using a WaterScout SM 100 Soil Moisture Sensor^®^ (Spectrum Technologies, Aurora, IL, USA). Initial plant phenotypic analyses were performed when soil moisture reached 20, 17, 14 and 8%. Based on the initial results we selected to further analyze phenotype of plants when 14% soil moisture was reached.

At the Institute of Agricultural Biology and Biotechnology, CNR (IBBA-CNR, Milan, Italy), plants (*lpa280-10* mutant and sister wt line 905) were germinated and grown in a phytotron under controlled conditions for a 13/11 h photoperiod and at a temperature of 26 /22°C. A triple randomized block design with six replicates of each genotype per condition was used. Plants grown for two weeks were subjected to irrigation withholding to impose water stress. Control plants were watered continuously. During the stress period, phenotypic data were collected every two days for two weeks. Based on the degree of stress symptoms, plants were subdivided into four classes: Non-stressed, plants with normal appearance; Lightly stressed, plants with epinastic cotyledonary leaves; Partially stressed, lightly wilted leaves; Severely stressed, strongly wilted leaves. A triple randomized block design with six replicates of each genotype per condition was used. At the end of the water stress treatment, watering was replenished thus allowing plants to recover from the stress. During the first three days of recovery plant phenotypes were recorded and after one-week recovery period, the biomass (dry weight, (DW)) of 10–12 plants for each treatment and genotype was measured.

### 2.2. Analysis of Symbiotic Phenotype

Nitrogenase activity was determined by the acetylene reduction assay (ARA, [32]) in detached nodulated roots from 10 biological replicates for each treatment. Root samples were placed into 160 mL sealed vials, substituting 2 mL of the air with acetylene in order to reach a 10% atmosphere concentration and incubated at room temperature. After 15 and 30 min, 1 mL of the gas of each vial was removed and analyzed in the gas chromatographer to measure ethylene concentration. Specific activity was expressed as nmol ethylene min^−1^ g^−1^ nodule dry weight (gNDW).

Relative water content (RWC), turgid weight (TW) and DW were determined on (1 cm diameter) leaf discs from three fully expanded trifoliate leaves from six biological replicates per treatment. Relative water content from common bean plants under control and water stress treatment was determined as described by Rosales et al., [33]. For RWC and TW leaf discs were incubated in distilled water at 4 °C overnight and then weighted; next the discs were oven dried at 70 °C for three days before determining the DW in an analytical scale.

The osmotic potential was measured as described by Castro-Camus et al., [34] with slight modifications. Essentially two leaf discs (1.3 cm diameter) per leaf, from an average of five biological replicates, were snap-frozen and subjected to five cycles of 1 min liquid-nitrogen freezing/10 min room temperature thawing. Subsequently, the samples were centrifuged through a perforated tube for 20 min at 14,000 rpm and 10 µL of the recovered liquid were used for osmolality measurement using a Vapor Pressure Osmometer (VAPRO^®^ Model 5600, ELITechGroup, Puteaux, France) following the manufacturer’s instructions.

### 2.3. Statistical Analysis

One-way analysis of variance (ANOVA) was applied to the different parameters using Tukey honest significant difference (HSD) test for mean separation at *p* = 0.05. In some cases, Brown-Forsythe heteroscedastic ANOVA was used with Tamhane T2 post hoc test for mean separation at *p* = 0.05.

### 2.4. Real-Time Quantitative PCR Analysis

Total RNA was isolated from 200 mg frozen nodules from BAT 93 or *lpa1* inoculated plants grown under control or water stress, using Trizol reagent (Life Technologies, Carlsbad, CA, USA) as reported [35]. Total RNA (2 µg) was used as a template to synthesize complementary DNA (cDNA) using the RevertAid minus first strand cDNA Synthesis Kit (Thermo Scientific, Waltham, MA, USA), according to the manufacturer’s instructions. Resulting cDNAs were then diluted and used to perform quantitative real-time polymerase chain reaction (qRT-PCR) analysis to determine the transcript levels of selected genes. Genomic DNA (gDNA) removal, cDNA synthesis and quality verification for qRT-PCR were performed as previously reported [35,36]. Reactions were run in a 96-well format plates with the 7300 Real-Time PCR System and 7300 System Software (Applied Biosystems, Foster City, CA, USA).

The expression of selected bacterial genes was assessed by qRT-PCR. Total RNA isolated from common bean nodules was used to synthesize bacteroids cDNA using the RevertAid minus first strand cDNA Synthesis kit (Thermo Scientific) and random hexamer primers (Fermentas, Copenhagen, Denmark), following the manufacturer's instructions.

The sequences of the oligonucleotide primers used for qRT-PCR amplification of each nodule and bacteroid gene were previously reported [36]. Three biological replicates with two technical replicates each were carried out for the determination of transcript level of each gene. Relative expression for each sample was calculated using the comparative threshold cycle (*C_t_)* method [35]. The *C_t_* value obtained after each reaction was normalized to the *C_t_* value of the *UBC9* (Phvul.006G110100) reference gene for nodule transcripts and the *rpoA* (RHE_CH01699) reference gene for bacteroid transcripts, whose expressions remained constant across the conditions. Student’s *t*-test was performed with a *p*-value cut-off of 0.05.

## 3. Results

### 3.1. The Symbiotic Nitrogen Fixation Phenotype and Nodule Gene Expression Profile of *lpa1* Plants

**Figure 1:**
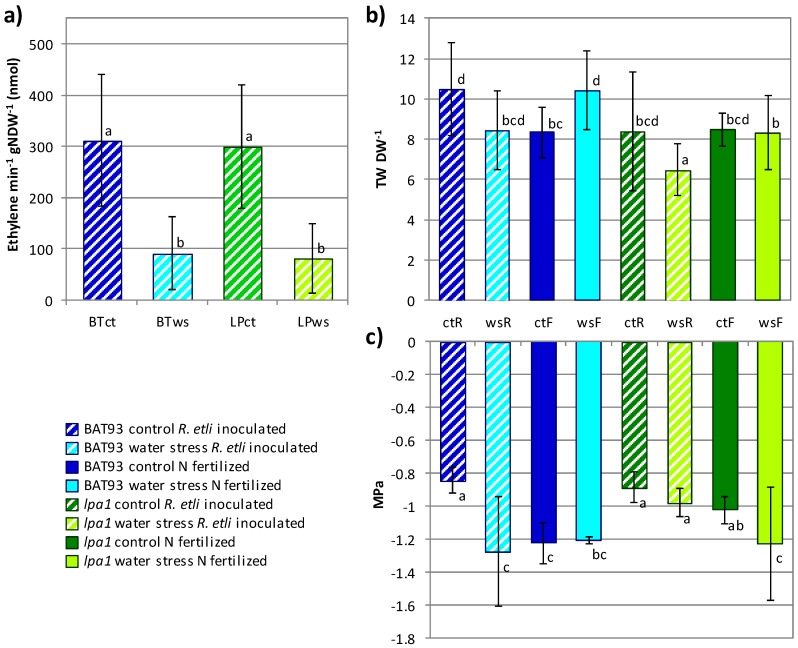
Nitrogenase activity measured by the acetylene reduction assay (ARA) (**a**), turgor weight (TW) to dry weight (DW) ratio (**b**) and osmotic potential (**c**) from inoculated or fertilized BAT 93 (BT, blue) and (*low phytic acid*) *lpa1* (LP, green) plants. Dashed and solid bars correspond to *Rhizobium etli* inoculated (R) and to fertilized (F) plants, respectively. Plants from the control treatment (ct, dark color) and from the water stress treatment (ws, light color) at 14% soil moisture were analyzed. Equal lowercase letters correspond to equal mean for analysis of variance (ANOVA) with Tukey honest significant difference (HSD) (**a**, **c**) or Tamhane T2 post hoc test (**b**). Bars represent ± Standard Deviation (SD). Nodule dry weight: gNDW.

Previous data obtained by our group indicated that the agronomic performance of the common bean *lpa1* biofortified mutant was not affected when grown in field conditions [17,18]. The biological N_2_-fixation, taking place in the legume-rhizobia symbiotic process, is relevant for sustainable agriculture allowing crop production in nutrient-limiting soils. In this work, we assessed the performance of SNF *lpa1* plants under laboratory (controlled environmental) conditions. Common bean *lpa1* and BAT 93 plantlets were inoculated with *R. etli* CE3 wt strain and plants were grown for three weeks. We observed that the nodule biomass (Figure S1) as well as nitrogenase activity, determined by the ARA assay (Figure 1a), were similar in the *lpa1* and BAT 93 plants. In addition, plants from both genotypes showed similar values for RWC (Figure S2), TW/DW ratio and osmotic potential (Figure 1b, c).

**Figure 2:**
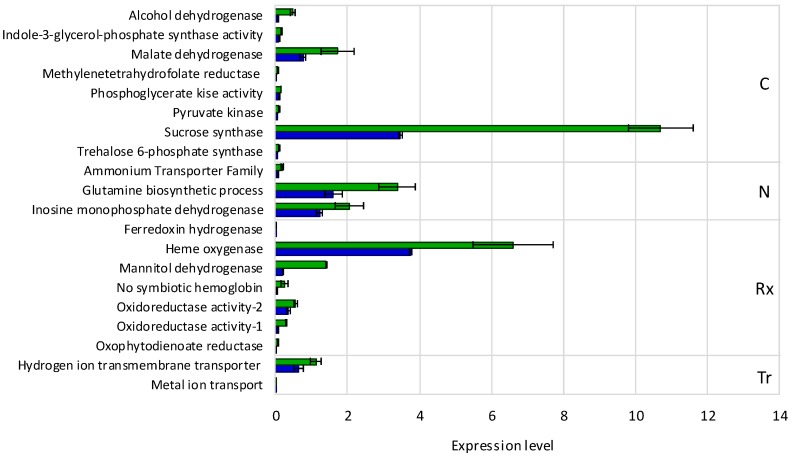
Relative expression level of nodule-function related genes in nodules of *lpa1* (green) and BAT 93 (blue) *R. etli* inoculated plants, grown under control conditions. The genes functional categories are: carbon metabolism (C), nitrogen metabolism (N), redox status (Rx) and transport (Tr). Bars represent ± SD. Relative expression for each sample was calculated using the comparative threshold cycle (C_t_) and the SUMO-conjugating enzyme UBC9 gene was used for normalization.

Based on our previous data on nodule gene expression profiles [36,37,38], in this work we analyzed transcript levels in mature nodules from *lpa1* mutant as compared to BAT 93 plants. For this we selected 20 genes that participate in relevant processes in mature nodules, such as: carbon and nitrogen metabolism, redox balance and transport, as shown in Figure 2. The trend of the level of gene expression was similar in nodules from both plant genotypes; genes showing low (i.e. ferredoxin hydrogenase, metal ion transport), medium (i.e. oxidoreductase activity, hydrogen ion transmembrane transporter) or high (i.e. sucrose synthase, heme oxygenase) expression levels in nodules from the analyzed genotypes were observed. However, the comparison of the expression level of each gene in nodules of *lpa1* vs. BAT 93 plants showed a significant increased expression of all the tested genes in the *lpa1* nodules (Figure 2).

### 3.2. Comparative Analysis of the Effect of Water Stress and the Response to Rehydration Recovery in *lpa1* vs. Wild Type Fertilized Plants

The *lpa* mutants from different plant species frequently display negative pleiotropic effects, however the *Arabidopsis atmrp5* mutant has been shown to be more tolerant to water deficit [25]. Tolerance to water scarcity is an important trait for common bean, thus in this work we performed a comparative analysis of the response to water stress of BAT 93 and *lpa1* mutant plants that showed no evident negative phenotypes [17,18]. Based on previous knowledge about a different water stress tolerance of nitrate-fed fertilized as compared to rhizobia-inoculated SNF common bean plants [7], in this work we analyzed the response to water stress of the *lpa1* mutant as compared to wt common bean plants grown in these two conditions differing in their N source.

**Figure 3:**
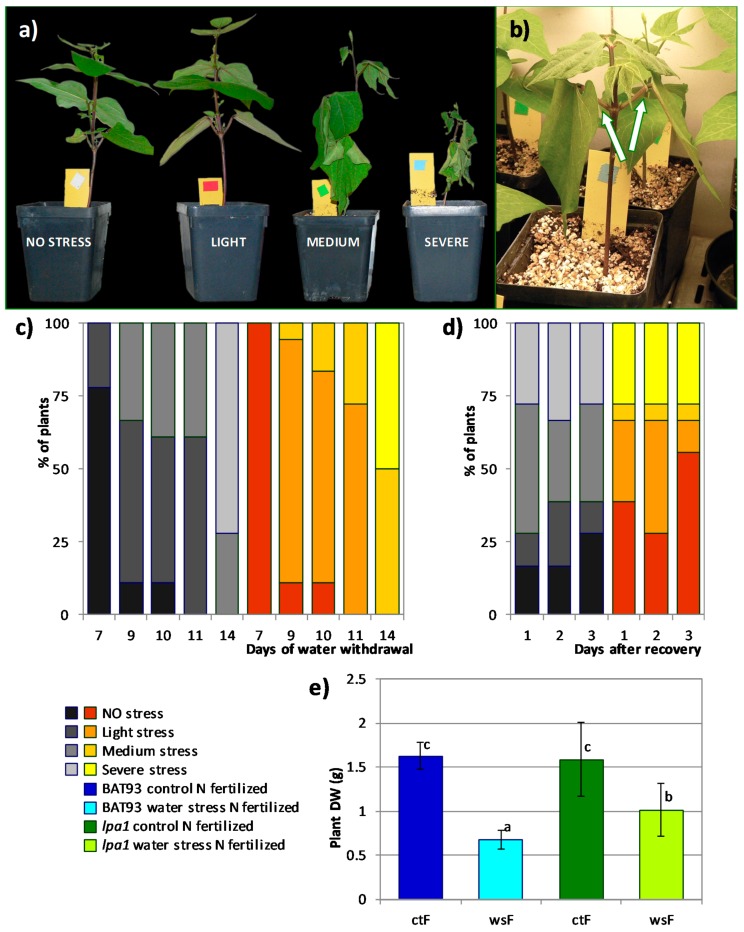
Effect of water withdrawal on two weeks old fertilized wild type (wt) and *lpa1* plants and response to one week of recovery. **(a)** Plants were classified, according to gradual level of water stress, into four phenotypical classes, showing no stress, light, medium or severe stress symptoms, as shown in the picture for wt plants as example. **(b)** Detail of a lightly stressed plant, showing epinastic cotyledonary leaves. **(c)** The percentage distribution of plants during the stress period, categorized in the four classes shown in (**a**) is indicated with darker to light colored bars, for wt (grey) and *lpa1* (orange) plants. **(d)** Plant phenotype during the first three days of recovery period, recording similar stress symptoms as those described in **(a)** and **(c)**. **(e)** Dry weight of control (ctF) and water stressed (wsF) plants at the end of one week of recovery, blue and green indicate wt and *lpa1* plants, respectively. Equal lowercase letters correspond to equal mean for ANOVA with Tamhane T2 post hoc test. Bars represent ± SD.

For the fertilized growth condition, we analyzed two weeks old wt and *lpa1* fertilized plants grown under controlled environmental conditions. Water stress symptoms of plants subjected to water withdrawal, were recorded every two days for a total of two weeks. The symptoms associated to water deployment were categorized in four phenotypical classes corresponding to no stress, light, medium or severe stress symptoms (Figure 3a, b). Water stress symptoms were recorded every two days for a total of two weeks; clear symptoms were observed after one week (Figure 3c). At this time point a small proportion (22%) of wt plants showed light symptoms of stress while all *lpa1* plants still displayed a normal phenotype. The different response between plant genotypes was confirmed in the following days. In fact, wt plants showed moderate to severe stress symptoms much earlier than *lpa1* plants, i.e. after 10 days of water withdrawal half of wt plants were lightly stressed and 39% showed medium stress symptoms as opposed to *lpa1* that included 72% lightly stressed and only 16% medium-stressed plants (Figure 3c). Fourteen days after water withdrawal, the majority (72%) of wt plants showed severe stress symptoms compared to only half of the *lpa1* plants (Figure 3c).

At the end of the water stress treatment, plants were re-hydrated and their stress symptoms were recorded during the recovery period, as shown in Figure 3d. After three days of recovery, 55% of the *lpa1* plants as compared to 28% of the wt plants showed a normal phenotype. By contrast, at this time point most wt plants (33%) and only 5% of *lpa1* plants still presented medium symptoms of stress. After one week of recovery the *lpa1* plants displayed a significantly different lower reduction in biomass (−36%) as compared to wt plants (−58%, Figure 3e). Our data indicate that *lpa1* mutant plants were less affected by water stress.

### 3.3. Comparative Analysis of the Effect of Water Stress in *lpa1* vs. BAT 93 Symbiotic Nitrogen Fixation Plants

**Figure 4:**
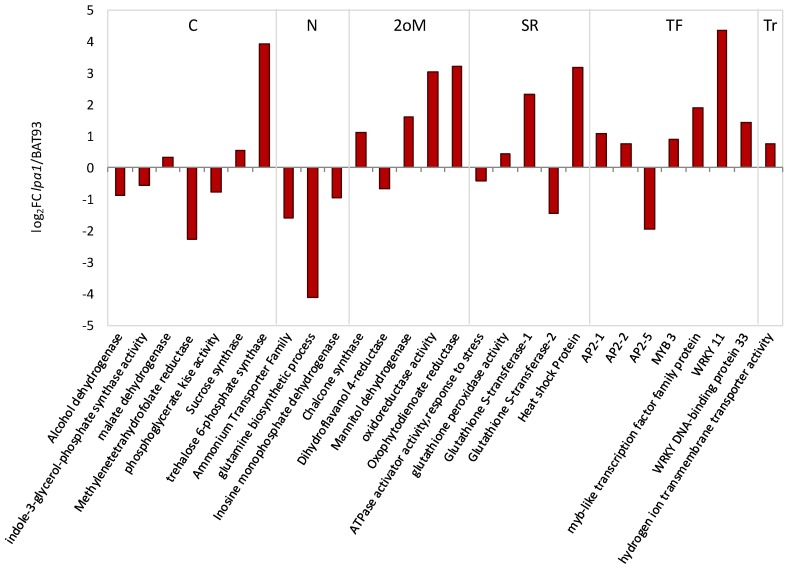
Relative expression level of nodule-function related genes in nodules of water stressed *lpa1* plants compared to BAT 93. For the water stress treatment, plants were analyzed at 14% soil moisture. Values refer to the log_2_ of the fold change ratio between *lpa1* and BAT93. The genes functional categories are: carbon metabolism (C), nitrogen metabolism (N), secondary metabolism (2oM), stress response (SR), transcription factors (TF) and transport (Tr).

We then extended the comparative analysis of the response to water stress to *lpa1* mutant vs. wt plants grown in SNF (Figure 1 and Figure 4). Water withdrawal was imposed to previously nodulated plants actively fixing N_2_; which were analyzed when 14% soil moisture was reached. This soil moisture value was selected based on results obtained in our initial trials that showed a very minor impairment in plants under higher percentage soil moisture (17%, 20%) and a very drastic effect in particular for the nodules at lower soil moisture (8%).

The osmotic potential (Ψ) values from the plants subjected to different treatments are shown in Figure 1c. In general, BAT 93 plants showed lower Ψ values as compared to *lpa1* plants. A notable difference was observed in SNF growth condition, where BAT 93 stressed plants showed significantly decreased Ψ as compared to irrigated plants and also as compared to SNF *lpa1* stressed plants. The RWC values for water stressed SNF plants showed no significant difference between both genotypes and also these were similar to values form control plants (Figure S1). Figure 1b shows the data on TW/DW determinations. Again, a notable difference was observed in SNF plants; the *lpa1* plants showed significantly lower TW/DW than the BAT 93 plants, especially under water stress. In fertilized condition the BAT 93 plants under water stress significantly increased their TW/DW ratio. As expected, the water stress resulted in a reduction of nitrogenase activity (determined by ARA) to a similar level in both genotypes (Figure 1a).

We performed nodule gene expression analysis in *lpa1* and BAT 93 SNF plants under water stress vs. control. For this, 54 genes were selected, including 20 genes related to nodule function shown in Figure 2 and other genes known to be expressed in common bean nodules from plants under abiotic stress [36,37,38]. Half of these genes were differentially expressed in water stress vs. control condition in both plant genotypes; their expression levels are shown in Table S1. The comparison of gene expression response to water stress in nodules from *lpa1* vs. BAT 93 plants is shown in Figure 4. A distinct expression profile was observed in nodule function-related genes (carbon and nitrogen metabolism functional categories) as compared to stress response related genes (from secondary metabolism, stress response, transcription factors and transport functional categories) between different genotypes. In *lpa1* nodules 2 out of 10 C/N metabolism genes showed lower expression level, while 4 out of 17 stress response related genes showed increased expression as compared to BAT 93 nodules under water stress conditions (Figure 4).

We determined the expression of selected SNF-related and stress responsive genes from bacteroids of *lpa1* as compared to BAT 93 plants subjected to water stress (Table 1). The expression of the transcriptional regulator NifA protein (*nifA)*, the activator of nitrogenase iron protein (*nifH)* in nitrogen-fixing bacteria, was moderately increased in *lpa1* bacteroids; however, this did not correlate with the decreased *nifH* gene expression. The *fixN*d gene, part of the *fixNOQP*d operon, encoding the symbiotic *cbb_3_* oxidase complex [39], showed highly increased expression in the *lpa1* bacteroids. In addition, two genes, the catalase (*katG)* and the hydrogen peroxide sensing transcriptional regulator protein (*oxyR)*, which respond to oxidative stress in different bacteria, showed a higher expression level in *lpa1* bacteroids.

## 4. Discussion

It is well known that phytic acid is not only an important molecule for seed phosphorus storage, but, together with its precursors (lower InsPs and *myo*-inositol) and its derivative molecules (InsP_7_ and InsP_8_ inositol pyrophosphates), it has an important role in regulation of different cell signaling and plant processes, including signal transduction, sugar signaling, storage and polar transport of auxin, membrane trafficking, abiotic and biotic stress response, phosphorus homeostasis, photomorphogenesis, chromatin modification and remodeling and mRNA nuclear export [21]. Indeed, in different crops *lpa* mutants affected in the phytic acid transporter were described to have negative pleiotropic effects, however this does not hold true for common bean as it possesses a paralog of the *PvMRP1* transporter, the *PvMRP2* gene. However, these two genes are not completely redundant in their function; in fact, this work shows that the absence of a functional *PvMRP1* transporter in the *lpa1* mutant confers phenotypic and molecular alterations in comparison to the wt. To our knowledge there is no information concerning the role of phytic acid in legume nodules. In this work, we extend the characterization of the common bean *lpa1* mutant, affected in the *PvMRP1,* with regard to its ability to establish the N_2_-fixation symbiosis and to respond to water stress.

We showed that plants from the *lpa1* genotype, grown under environmentally controlled condition, are able to establish an efficient SNF with *R. etli* CE3, presenting equal nodule biomass and nitrogenase activity as compared to its wt parental genotype (Figure 1 and Figure S1). In addition, *lpa1* nodules showed high expression of C/N metabolism and stress response-related genes. The *lpa1* capacity for efficient SNF could be relevant for crop production of this nutritionally improved genotype through sustainable agricultural practices.

The drought response of *lpa1* mutants from different plant species has been poorly studied, something that may be related to their, often observed, stunted growth phenotype under standard conditions [21]. For example, the maize *lpa1-1* mutant, affected in the *ZmMRP4* (*Zea mays* multidrug resistance-associated protein 4) transporter gene, orthologue to the common bean *PvMRP1* gene, was described as more sensitive to drought stress in the field [40] and this negative pleiotropic effect could be associated with an alteration of the mature root system, described for the allelic *lpa1-7* mutant [41]. On the other side, the *Arabidopsis mrp5-1* mutant shows reduced water loss from detached rosette leaves, reduced transpiration rate, improved water use efficiency and enhanced drought stress tolerance that were attributed to a reduced stomatal aperture under light. Moreover, *mrp5-1* stomata closure showed reduced sensitivity to calcium and abscisic acid [25]. It was shown that the AtMRP5 (*Arabidopsis thaliana* multidrug resistance-associated protein 5) transporter is able to modulate anion and calcium-channels activity in guard cells [42].

In this work, we analyzed the response of common bean *lpa1* plants to water stress, including fertilized plants—sufficient nitrate as the N_2_ source—and *R. etli* inoculated plants—fixed-N_2_ as N_2_ source—. The RWC values we obtained for common bean SNF plants under water stress vs. control treatments were not significantly different. However, fertilized plants under water stress of both *lpa1* and BAT 93 genotypes showed significant lower RWC values (88% and 86%) than control plants (93% and 92%) (Figure S2). These results are in agreement with what Lodeiro et al., [7] and Kirova et al., [6] described for common beans and soybeans. However, it is difficult to estimate a reliable value of RWC because often different common bean varieties are able to change the leaf water status during the day in a way not always related to its drought tolerance/susceptibility (see for example [33]). This fact influences in particular the fresh weight, while TW and DW depends mainly by the leaf tissue morphology and compositions, making them more reliable in estimating bean response to water stress. For this reason, we also determined the TW/DW ratio from the leaves of plants from the different treatments (Figure 1). The TW/DW value displayed by fertilized BAT 93 plants under water stress was the highest, while there was no difference in TW/DW value between stressed and control fertilized *lpa1* plants, this value was similar to that from control wt plants. However, when symbiosis took place, *lpa1* plants showed significantly lower TW/DW under water stress, indicating a possible synergic effect between bacteria and genotype (Figure 1). Martinez et al., [4] reported a negative correlation between drought resistance index (DRI) and TW/DW. Drought resistance index was created to assess drought resistance independently from yield potential and phenology effects [43]. The lower value of TW/DW measured in SNF *lpa1* than in wt SNF plants should indicate a higher DRI of *lpa1* than BAT 93 plants, identifying, at the end, a better performance of *lpa1* plants under water stress.

Our phenotypic analysis of fertilized plants subjected to water stress and recovery upon re-hydration, revealed milder water stress symptoms, as well as significantly higher plant biomass in *lpa1* as compared to wt plants (Figure 3). These data would indicate a better performance of fertilized *lpa1* plants to water stress.

Several studies have documented the different processes that are affected in SNF legumes growing under water stress conditions; these include nitrogen fixation, nodule oxygen permeability and regulation of carbon and nitrogen metabolism [9,13]. In agreement with previous knowledge, the nitrogenase activity was affected by water stress but no significant difference was observed between activity levels of *lpa1* and wt genotypes (Figure 1). The latter could be related to the ARA method used for determining nitrogenase activity that is a traditional assay still largely used but that has been shown to be not very precise [8]. A more accurate determination of nitrogenase activity together with the analysis of larger plant populations that may compensate the observed high variability among individual plants could help revealing possible differences in SNF among the mutant and wt genotypes analyzed here.

We assessed possible effects of water stress on C/N metabolism of common bean nodules by evaluating transcript levels of genes known to participate in these processes and to respond to stress [32,33,34]. Notably, we observed clear differences in gene expression in nodules from *lpa1* vs. BAT 93 plants both under water stress and under control conditions (Figure 2 and Figure 4 and Table S1). The *lpa1* nodules showed an increased expression of nodule-function genes (C/N metabolism or redox balance) in control plants (Figure 2). Our preliminary transcriptomic microarray analysis comparing leaf gene expression from fertilized *lpa1* vs. wt common bean plants showed similar results, regarding higher gene expression levels in *lpa1* leaves [44]. Our interpretation of these data is that the *lpa1* mutant is, in some way, already prepared to cope with stress, the elevated gene expression in control nodules could be sufficient for maintaining adequate metabolic/physiological conditions for responding when adverse environmental conditions appear. Interestingly, most of the selected C/N metabolism genes were upregulated to higher levels in water stressed BAT 93 as compared to *lpa1* nodules (Figure 4). It is known that sucrose synthase is one of the first enzymes to be affected by water stress, this is followed by a decrease in malate content, thus a reduction of carbon supply to bacteroids and a consequent reduction in nitrogen fixation [8,10,12]. The gene coding for nodule sucrose synthase is regulated at the transcriptional and post-transcriptional levels [13]. It is interesting to note that in control condition sucrose synthase is more expressed (three-fold) in the *lpa1* plants than in the BAT 93 ones, while under water stress conditions its expression is decreased to a similar level in both genotypes that showed similar decrease in nitrogenase activity (Table S1, Figure 1). Our interpretation is that the constitutive higher expression of sucrose synthase in control *lpa1* plants could favor the accumulation of dicarboxylates in the nodules thus preparing the plants to stand water stress. In the *lpa1* nodules, as compared to the BAT 93 ones, trehalose 6-P synthase was more expressed (two-fold) and in response to water stress its expression increased up to 15-fold. It is known that in *Arabidopsis*, overexpression of the trehalose synthase gene (*AtTPS1)* caused only low level of trehalose accumulation but this was sufficient to confer desiccation tolerance [45]. Because the increase of trehalose concentration was too low to explain an effect as osmoprotector, trehalose has been proposed as a signal molecule activating stress tolerance [45,46]. We determined the expression level of stress-related genes in nodules from control and water stressed plants; these genes were assigned to the functional categories of secondary metabolism, stress response, transport and transcription factors gene families known to be stress-responsive [36,47,48]. As expected, these genes were upregulated in water stressed nodules, being the *lpa1* stressed nodules those with the highest gene expression (Figure 4, Table S1).

The effects of water stress in bacteroid gene expression was also assessed by determining the expression level of SNF and stress response-related genes; notable differences were observed in levels of gene expression from bacteroids of *lpa1* vs. BAT 93 nodules (Table 1). A similar effect as that observed for nodule stress related genes was observed for two bacteroids stress-responsive genes (*katG* and *oxyR*) whose expression was increased in bacteroids from *lpa1* water stressed plants. Notably the expression of *fixN*d, was eight-fold higher in bacteroids from *lpa1* plants as compared to bacteroids from wt plants under water stress. The responses of nodule function to drought and other stresses is related to the closure of oxygen diffusion barrier in the nodule cortex, that results in a decrease in oxygen availability for bacteroidal respiration leading to a lack of energy for SNF [8]. The overexpression of *cbb_3_* oxidase genes have been related to a higher bacteroid respiratory capacity [49]. We propose that the increased expression of *cbb_3_* in water stressed *lpa1* bacteroids could be related to a better oxygen diffusion and bacteroidal respiration that leads to enhanced SNF. The differential gene expression observed in nodule and bacteroids of SNF *lpa1* plants could be relevant for improved water stress response.

Previous knowledge from *Arabidopsis* has defined the function of MRPs transporters in exporting InsP_6_ from the cytosol to the vacuole [42]. In mutants affected in this transporter activity, increased concentration of InsP_6_ in the cytosol is expected. However, these mutants show a strong *lpa* phenotype in the seeds, due to a negative feedback on the InsP_6_ biosynthetic genes, as shown in the common bean *lpa1* and in the *Arabidopsis mrp5* mutants [19,50] but also to the accumulation of the InsP_7_ and InsP_8_ inositol pyrophosphates [51], as demonstrated in the *Arabidopsis mrp5* and in the maize *mrp4* mutants. Moreover, a possible degradation activity by phytases was also hypothesized in the common bean *lpa1* seeds [19]. It is not clear what happens in this kind of mutants in tissues other than the seeds. It can be hypothesized that altered cytosolic InsP_6_ concentration may affect cell metabolism. Because InsP_6_ has important roles in regulating cell signaling, including sugar signaling [21] one can envisage that an altered cytosolic InsP_6_ concentration in *lpa1* nodule cells can, in turn, modify signal transduction pathways that would result in global gene expression changes. Such global regulatory modifications could affect—positively or negatively—several cellular processes such as the response to water deficit.

Different features reported here for the *lpa1* common bean mutant under water stress, such as milder water stress symptoms and increased biomass in fertilized plants as well as lower TW/DW value indicating a higher DRI, higher nodule-function gene expression in prior control condition, higher stress-related gene expression in nodules and bacteroids as well as higher symbiotic *cbb_3_* oxidase expression in SNF plants may be relevant for better coping to the stress of this nutritionally improved genotype. Integrated research projects regarding plant phenotyping in water stress field conditions are required for a conclusive evaluation of the tolerance to drought of the common bean *lpa1* mutant.

## Figures and Tables

**Table 1:** *Rhizobium etli* bacteroids gene expression from *low phytic acid* (*lpa1)* and BAT 93 plants under water stress treatment. (*p* < 0.05).

Gene	Annotation	*lpa1*	BAT 93	Fold Change *lpa1*/BAT93 (log_2_)
*nifA*	Transcriptional regulator NifA protein	0.128	0.075	0.778
*nifH*	Nitrogenase, iron protein	6.485	13.661	−1.075
*fixN*d	*cbb_3_* oxidase	0.150	0.018	3.037
*katG*	Catalase	0.437	0.200	1.126
*oxyR*	Hydrogen peroxide sensing transcriptional regulator protein	0.042	0.006	2.899

## Supplementary Materials

The following are available online at http://www.mdpi.com/2073-4425/9/2/99/s1, Figure S1. Nodule biomass of wt and *lpa1* from SNF plants under control and water stress treatment. Figure S2. Relative water content (RWC) from leaves of fertilized and SNF wt or *lpa1* plants under control and water stress treatments. Table S1. Differentially expressed genes in nodules of *lpa1* and BAT 93 *R. etli* inoculated plants grown under control or water stress conditions.
