# Supplementary material for: Characterization of the Symbiotic Nitrogen-Fixing Common Bean Low Phytic Acid (lpa1) Mutant Response to Water Stress
Source: Genes (Basel). 2018 Feb 15;9(2):99. doi: 10.3390/genes9020099 (PMC5852595; doi:10.3390/genes9020099)
Supplement: Supplementary file 1 [file genes-09-00099-s001.docx]

**Figure S1.** Nodule biomass of wt and *lpa1* from SNF plants under control and water stress treatment. Plants from the control treatment (ct, dark color) and from the water stress treatment (ws, light color) at 14% soil moisture were analyzed. Equal lowercase letters correspond to equal mean for ANOVA with Tukey HSD. Bars represent ± SD.

**Figure S2.** Relative water content (RWC) from leaves of fertilized and SNF wt or *lpa1* plants under control and water stress treatments. Dashed and solid bars correspond to R. etli inoculated (R) and to fertilized (F) plants, respectively. Plants from the control treatment (ct, dark color) and from the water stress treatment (ws, light color) at 14% soil moisture were analyzed. Equal lowercase letters correspond to equal mean for ANOVA with Tamhane T2 post hoc test. Bars represent ± SD.

**Table S1.** Differentially expressed genes in nodules of *lpa1* and BAT93 *R. etli* inoculated plants grown under control or water stress conditions.

Plants were analyzed when soil moisture reached 14%. Values represent expression level as determined by qRT-PCR (normalized data with *UBC* housekeeping gene). The fold change ratio log_2_ (≥0.5) between water stress and control condition is shown (*p*≤ 0.05).

|  | ***lpa1*** | | | **BAT 93** | | |
| --- | --- | --- | --- | --- | --- | --- |
|  | **Control** | **Water stress** | **Expression Ratio Ws/C FC (log_2_)** | **Control** | **Water stress** | **Expression Ratio Ws/C FC (log_2_)** |
| Alcohol dehydrogenase | 0.487 | 0.077 | -2.657 | 0.074 | 0.141 | 0.935 |
| indole-3-glycerol-phosphate synthase activity | 0.176 | 0.083 | -1.091 | 0.091 | 0.123 | 0.433 |
| Malate dehydrogenase | 1.714 | 0.901 | -0.929 | 0.769 | 0.714 | -0.108 |
| Methylenetetrahydrofolate reductase | 0.059 | 0.020 | -1.573 | 0.020 | 0.094 | 2.262 |
| Phosphoglycerate kinase activity | 0.153 | 0.053 | -1.528 | 0.114 | 0.090 | -0.333 |
| Sucrose synthase | 10.705 | 2.224 | -2.267 | 3.472 | 1.515 | -1.197 |
| Trehalose 6-phosphate synthase | 0.100 | 0.698 | 2.805 | 0.049 | 0.046 | -0.085 |
| Ammonium Transporter Family | 0.187 | 0.027 | -2.784 | 0.084 | 0.083 | -0.012 |
| Glutamine biosynthetic process | 3.384 | 0.042 | -6.336 | 1.605 | 0.726 | -1.144 |
| Inosine monophosphate dehydrogenase | 2.038 | 0.402 | -2.341 | 1.219 | 0.772 | -0.660 |
| Hydrogen ion transmembrane transporter activity | 1.127 | 0.829 | -0.444 | 0.633 | 0.491 | -0.368 |
| Chalcone synthase | 0.003 | 0.002 | -1.138 | 0.002 | 0.001 | -1.100 |
| Dihydroflavanol 4-reductase | 0.235 | 0.116 | -1.014 | 0.037 | 0.183 | 2.323 |
| Mannitol dehydrogenase | 1.410 | 4.287 | 1.604 | 0.192 | 1.405 | 2.870 |
| Oxidoreductase activity | 0.306 | 3.097 | 3.338 | 0.075 | 0.377 | 2.322 |
| Oxophytodienoate reductase | 0.082 | 0.454 | 2.475 | 0.020 | 0.050 | 1.282 |
| ATPase activator activity, response to stress | 0.207 | 1.398 | 2.759 | 0.106 | 1.873 | 4.150 |
| Glutathione peroxidase activity | 0.571 | 0.432 | -0.404 | 0.370 | 0.317 | -0.224 |
| Glutathione S-transferase-1 | 2.904 | 9.145 | 1.655 | 0.246 | 1.845 | 2.904 |
| Glutathione S-transferase-2 | 1.255 | 4.123 | 1.716 | 0.862 | 11.367 | 3.721 |
| HSP | 0.420 | 10.751 | 4.679 | 0.232 | 1.199 | 2.368 |
| AP2-1 | 0.001 | 0.003 | 1.688 | 0.000 | 0.001 | 2.807 |
| AP2-2 | 0.022 | 0.046 | 1.080 | 0.012 | 0.028 | 1.258 |
| AP2-5 | 0.001 | 0.003 | 1.893 | 0.002 | 0.010 | 2.751 |
| MYB-3 | 0.412 | 0.932 | 1.176 | 0.118 | 0.496 | 2.075 |
| WRKY 11 | 0.007 | 0.036 | 2.334 | 0.001 | 0.002 | 1.585 |
| WRKY DNA-binding protein 33 | 0.383 | 1.642 | 2.101 | 0.173 | 0.608 | 1.814 |
